# Transcriptome-wide Dynamics of m^6^A mRNA Methylation During Porcine Spermatogenesis

**DOI:** 10.1016/j.gpb.2021.08.006

**Published:** 2021-09-17

**Authors:** Zidong Liu, Xiaoxu Chen, Pengfei Zhang, Fuyuan Li, Lingkai Zhang, Xueliang Li, Tao Huang, Yi Zheng, Taiyong Yu, Tao Zhang, Wenxian Zeng, Hongzhao Lu, Yinghua Lv

**Affiliations:** 1 Key Laboratory for Animal Genetics, Breeding and Reproduction of Shaanxi Province, College of Animal Science and Technology, Northwest A&F University, Yangling 712100, China; 2 School of Biological Science and Engineering, Shaanxi University of Technology, Hanzhong 723001, China; 3 College of Chemistry and Pharmacy, Northwest A&F University, Yangling 712100, China

**Keywords:** *N*^6^-methyladenosine, Spermatogonial stem cell, Spermatogenesis, *SETDB1*, Pig

## Abstract

**Spermatogenesis** is a continual process that occurs in the testes, in which diploid **spermatogonial stem cells** (SSCs) differentiate and generate haploid spermatozoa. This highly efficient and intricate process is orchestrated at multiple levels. ***N*^6^-methyladenosine** (m^6^A), an epigenetic modification prevalent in mRNAs, is implicated in the transcriptional regulation during spermatogenesis. However, the dynamics of m^6^A modification in non-rodent mammalian species remains unclear. Here, we systematically investigated the profile and role of m^6^A during spermatogenesis in **pigs**. By analyzing the transcriptomic distribution of m^6^A in spermatogonia, spermatocytes, and round spermatids, we identified a globally conserved m^6^A pattern between porcine and murine genes with spermatogenic function. We found that m^6^A was enriched in a group of genes that specifically encode the metabolic enzymes and regulators. In addition, transcriptomes in porcine male germ cells could be subjected to the m^6^A modification. Our data show that m^6^A plays the regulatory roles during spermatogenesis in pigs, which is similar to that in mice. Illustrations of this point are three genes (***SETDB1***, *FOXO1*, and *FOXO3*) that are crucial to the determination of the fate of SSCs. To the best of our knowledge, this study for the first time uncovers the expression profile and role of m^6^A during spermatogenesis in large animals and provides insights into the intricate transcriptional regulation underlying the lifelong male fertility in non-rodent mammalian species.

## Introduction

*N*^6^-methyladenosine (m^6^A) is a ubiquitous epigenetic marker in mammalian mRNAs [1]. As the first reversible RNA modification, m^6^A is installed by methyltransferase (METTL3, METTL14, and WTAP) [2], and reversed by demethylases FTO and ALKBH5 [3], [4]. m^6^A is recognized by YTHDF1, YTHDF2, eIF3, and others [5], [6]. Of these m^6^A-reader proteins, YTHDF2 recognizes m^6^A in mRNAs and targets the transcripts triggering the rapid degradation of m^6^A-containing mRNAs, whereas YTHDF1 and eIF3 bind to m^6^A-containing transcripts and promote the translation [2]. The critical roles of m^6^A involved in numerous biological processes, such as maternal mRNA clearance, DNA repair, embryonic development, sex determination, and spermatogenesis [7], [8], [9], [10], [11], [12], have been revealed in recent studies using knockout mouse models.

The testis offers lifelong male fertility by producing billions of sperm daily [13]. Sperm are derived from spermatogonial stem cells (SSCs), which undergo self-renewal divisions to maintain the stem cell pool or differentiation to generate progenitors and spermatogonia (SPG). The differentiated SPG further develop into preleptotene spermatocytes, which undergo a last round of DNA replication before entering meiosis. Through meiosis, haploid round spermatids (RS) are generated with dramatic changes in morphology and physiology [14], [15]. This highly organized process, named spermatogenesis, requires timely coordinated gene expression at the transcriptional and post-transcriptional levels [16]. Spermatogenesis also involves unique characteristics, such as premade transcripts, high levels of alternative splicing, and decreased or ceased transcriptional activity at onset of meiotic prophase I and late spermiogenesis [17]. Note that m^6^A modification that mediates mRNA splicing, stability, and translation [18], [19], [20] is involved in spermatogenesis, as demonstrated by loss-of-function studies for m^6^A writers [11], [21], erasers [4], [22], and readers [23] in mice.

Pigs (*Sus scrofa*), which are responsible for more than one third of meat produced worldwide, are important to global living demands and food security [24]. In addition, pigs are an excellent large animal model in biomedical research, owing to their similarities to humans in anatomy, physiology, and genetics. Although the mechanisms for spermatogenesis have been investigated extensively in mice, they remain poorly understood in pigs, and the roles of m^6^A in porcine spermatogenesis remain largely elusive. Here, we isolated porcine SPG, pachytene spermatocytes (PS), and RS, and performed the m^6^A affinity purification followed by m^6^A sequencing (m^6^A-seq) and RNA sequencing (RNA-seq). By analyzing the abundance of m^6^A and its roles in porcine spermatogenesis, we highlight for the first time the magnitude of m^6^A in transcriptional regulation in porcine spermatogenesis, thereby laying the foundation for future endeavors to link m^6^A to research and therapy for male infertility.

## Results

### Enrichment and characterization of porcine male germ cells

**Figure 1:**
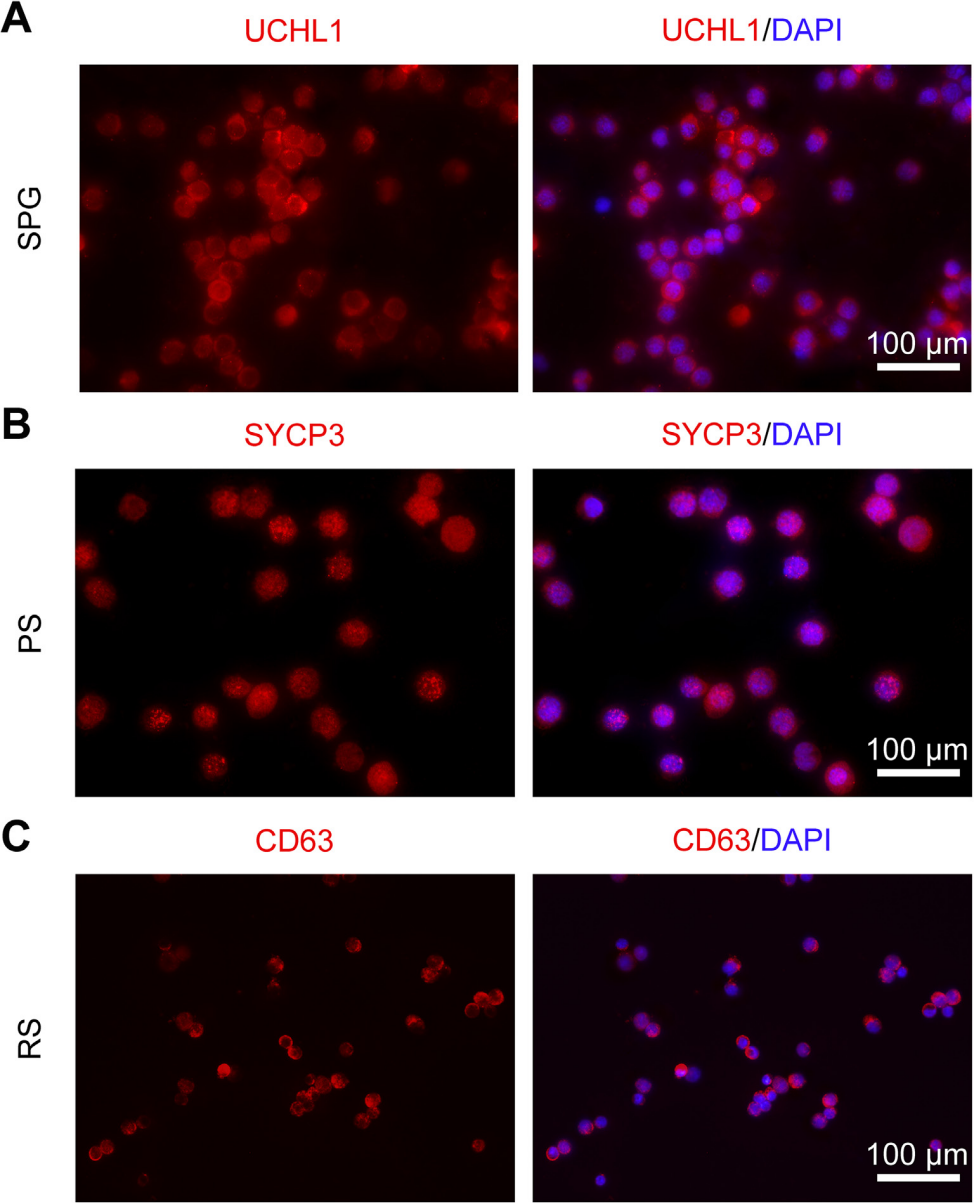
**Identification of porcine male germ cells** **A.** Immunocytochemistry showing the expression of UCHL1 in the SPG. **B.**  Immunocytochemistry showing the expression of SYCP3 in the freshly isolated PS. **C.** Immunocytochemistry showing the expression of CD63 in the RS. SPG, spermatogonium; PS, pachytene spermatocyte; RS, round spermatid; UCHL1, ubiquitin carboxyl-terminal esterase L1; SYCP3, synaptonemal complex protein 3; CD63, CD63 Molecule; DAPI, 4′,6-diamidino-2-phenylindole. Scale bar, 100 µm.

To obtain the transcriptome-wide map of m^6^A during spermatogenesis, we first isolated SPG, PS, and RS from porcine testes using STA-PUT velocity sedimentation. The purity of the isolated SPG, PS, and RS was determined by several biomarkers. Immunocytochemical analysis showed that the isolated SPG, PS, and RS were positive for ubiquitin carboxyl-terminal esterase L1 (UCHL1), synaptonemal complex protein 3 (SYCP3), CD63 Molecule (CD63), respectively (Figure 1A–C). In addition, the nuclei of the isolated SPG, PS, and RS were around 7–8 µm, 12–13 µm, and 5 µm in diameter, respectively (Figure 1A–C). These results suggest that the freshly isolated germ cells were SPG, PS, and RS with biochemical and nuclear characteristics.

### m^6^A-seq analysis of porcine male germ cells

**Figure 2:**
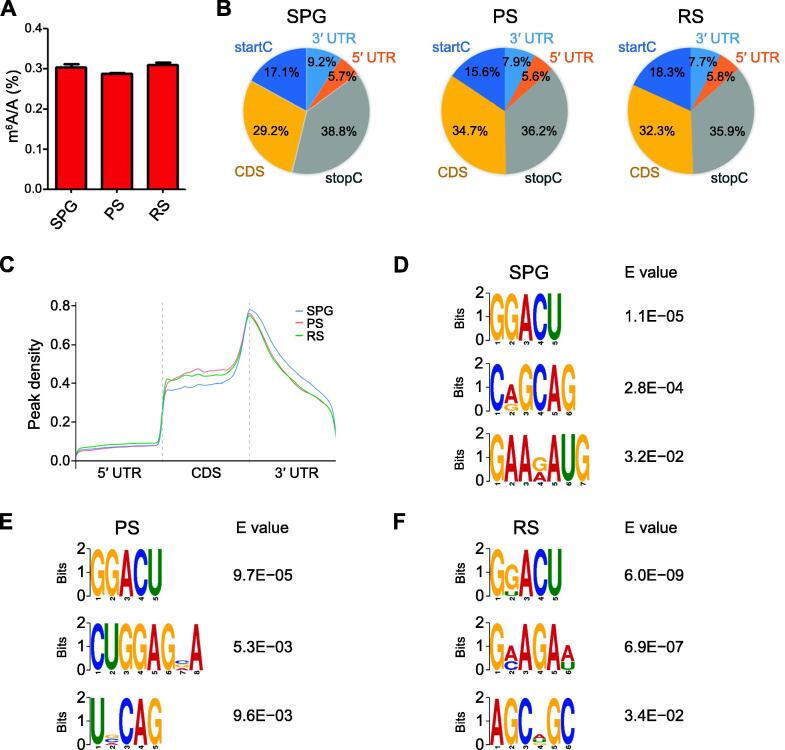
**Distribution pattern of m^6^A peaks along transcripts** **A.** LC-MS/MS analysis of m^6^A percentage relative to adenosine in SPG, PS, and RS. **B.** m^6^A peak distribution within different gene contexts: startC, CDS, stopC, 3′ UTR, and 5′ UTR. **C.** Accumulation of m^6^A-IP reads along transcripts in SPG, PS, and RS. Each transcript is divided into three parts: 5′ UTR, CDS, and 3′ UTR. **D.**–**F.** The top 3 used motifs among m^6^A peaks in SPG (D), PS (E), and RS (F). LC-MS/MS, liquid chromatography-tandem mass spectrometry; startC, start codon; CDS, coding sequence; stopC, stop codon; UTR, untranslated region.

To elucidate the m^6^A methylome in different stages of spermatogenesis, the liquid chromatography-tandem mass spectrometry (LC-MS/MS) was performed to quantify the changes of m^6^A modification in the isolated germ cells (Figure 2A). The m^6^A was presented in all mRNAs of the tested male germ cells, and the level of m^6^A remained relatively stable (∼ 0.3%) during the developmental stages. To further uncover the dynamics of m^6^A, the m^6^A-seq was performed and the locations of m^6^A peaks along the transcripts were determined. We found that m^6^A peaks were highly enriched near the start codon (startC), coding sequence (CDS), and stop codon (stopC) in the germ cells, but there were some differences among the three stages of germ cells (Figure 2B). The m^6^A peaks near the startC were 17.1% in SPG, 15.6% in PS, and 18.3% in RS (Figure 2B). m^6^A peaks near the CDS increased 5.5% from SPG to PS, followed by a 2.4% drop from PS to RS (Figure 2B). Furthermore, the abundance of m^6^A peaks near the stopC decreased 2.6% in PS (36.2%; Figure 2B) and then stabilized in RS (35.9%; Figure 2B).

The distribution of m^6^A in the whole transcriptome was validated by the m^6^A reads along transcripts. Consistent with the distribution of m^6^A peaks, m^6^A reads were distributed throughout the mRNA transcripts, in which the reads increased in the CDS and reached the peak at the 3′ UTR (Figure 2C). Specifically, in the CDS, the density of m^6^A reads in PS was higher than that in RS, followed by in SPG (Figure 2C). In addition, the density of m^6^A reads in the 3′ UTR of SPG was higher than that in PS and RS (Figure 2C). Together, the results reveal that m^6^A is dynamic in porcine male germ cells, which suggests its critical roles during spermatogenesis.

To determine whether the RRACH is the m^6^A consensus sequence in porcine germ cells, we analyzed the 1000 most significant peaks. The GGACU was a top motif in all tested samples (Figure 2D–F), suggesting that the RRACH motif adopted in porcine spermatogenesis is conserved in pigs and mice [11]. It is important to note that as a top motif in SPG (Figure 2D), PS (Figure 2E), and RS (Figure 2F), the GGACU is an m^6^A-modified sequence prevalent in porcine male germ cells.

### m^6^A-enriched genes are involved in important biological processes

**Figure 3:**
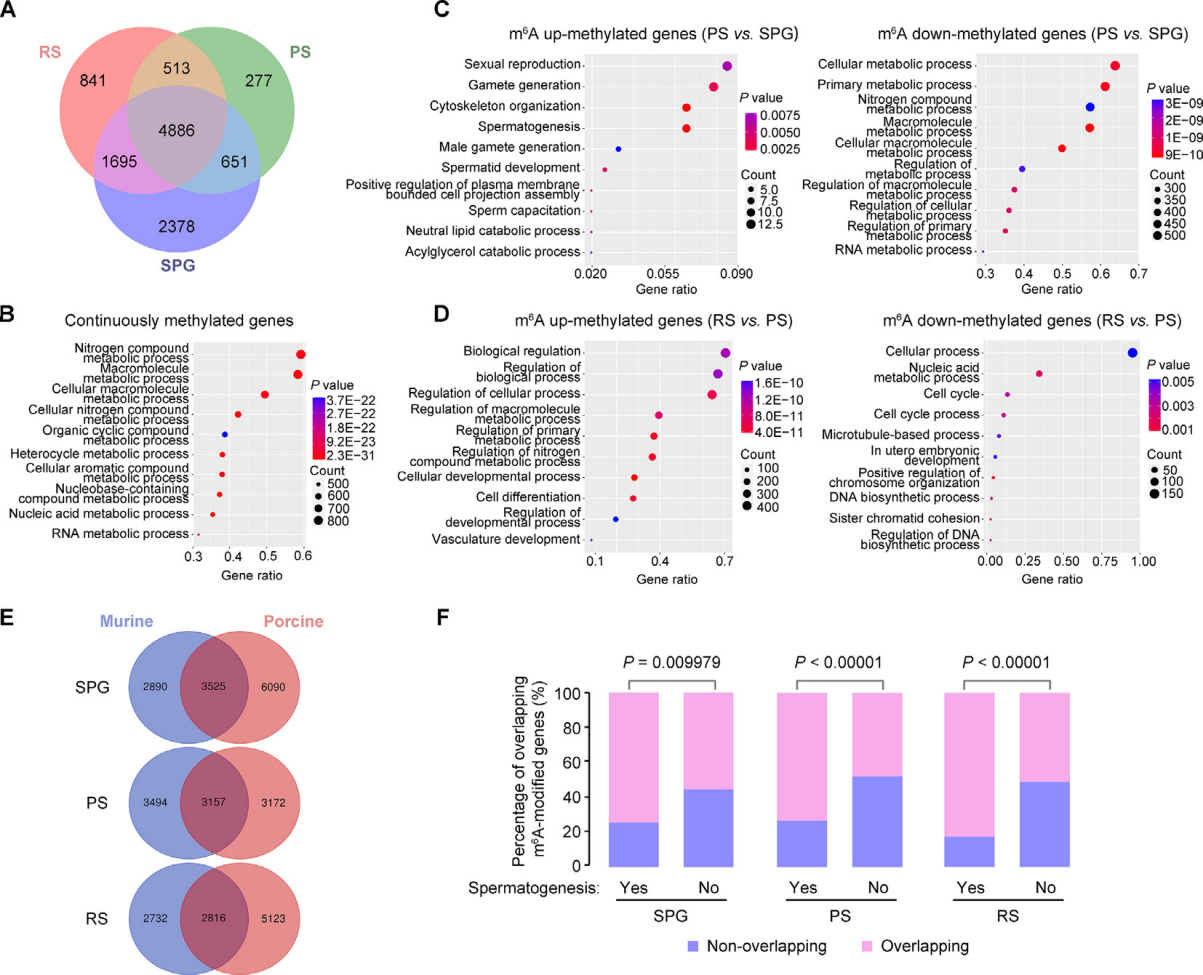
**m^6^A modification pattern during porcine spermatogenesis** **A.** Venn diagram showing the pattern of m^6^A-modified genes in SPG, PS, and RS. **B.** The enriched biological processes of continuously methylated genes during spermatogenesis by GO analysis. **C.** The enriched biological processes of DMGs between PS and SPG by GO analysis. **D.** The enriched biological processes of DMGs between RS and PS by GO analysis. Each dot plot shows gene ratio values of the top 10 significant enrichment terms. **E.** Venn diagram showing the overlapping m^6^A-modified genes between murine and porcine in SPG, PS, and RS. **F.** Different proportions of the overlapping methylated genes related or unrelated to spermatogenesis in murine and porcine in SPG, PS, and RS. The *P* value of such difference was calculated with the Chi-square test. GO, Gene Ontology; DMG, differentially methylated gene.

We discovered 11,241 methylated genes in porcine male germ cells. Of these, 2378, 277, and 841 methylated genes were exclusively expressed in SPG, PS, and RS, respectively (Figure 3A). Gene Ontology (GO) biological process analysis revealed that the 4886 continuously methylated genes were mostly involved in metabolic processes (Figure 3B). We then analyzed the genes containing altered m^6^A peaks (fold change ≥ 2, *P* ≤ 10E−5) to uncover more insights into m^6^A in porcine spermatogenesis. Results showed that 692 and 3662 genes were up- and down-methylated in PS, respectively, when compared to SPG; 3058 and 884 genes were up- and down-methylated in RS, respectively, when compared to PS (Table S1). GO biological process analysis revealed that up-methylated genes in PS (*vs.* SPG) were involved in spermatogenesis, whereas down-methylated genes were mostly involved in metabolic processes (Figure 3C). In addition, the up-methylated genes in RS (*vs.* PS) participated in developmental and metabolic processes, and down-methylated genes were mostly involved in the regulation of chromosome organization, nucleic acid metabolic, and microtubule-based process (Figure 3D).

Next, we compared the m^6^A-modified genes between porcine and mouse germ cells [11]. A total of 6090, 3172, and 5123 m^6^A-modified genes were uniquely found in porcine SPG, PS, and RS, respectively, whereas 3525, 3157, and 2816 m^6^A-modified genes were shared by both murine and porcine SPG, PS, and RS, respectively (Figure 3E). Notably, these overlapping m^6^A-methylated transcripts were preferentially enriched and reported to be essential for mouse spermatogenesis [11] (Figure 3F), indicating that m^6^A mediates conserved processes in male germ cells.

### Gene expression during spermatogenesis

**Figure 4:**
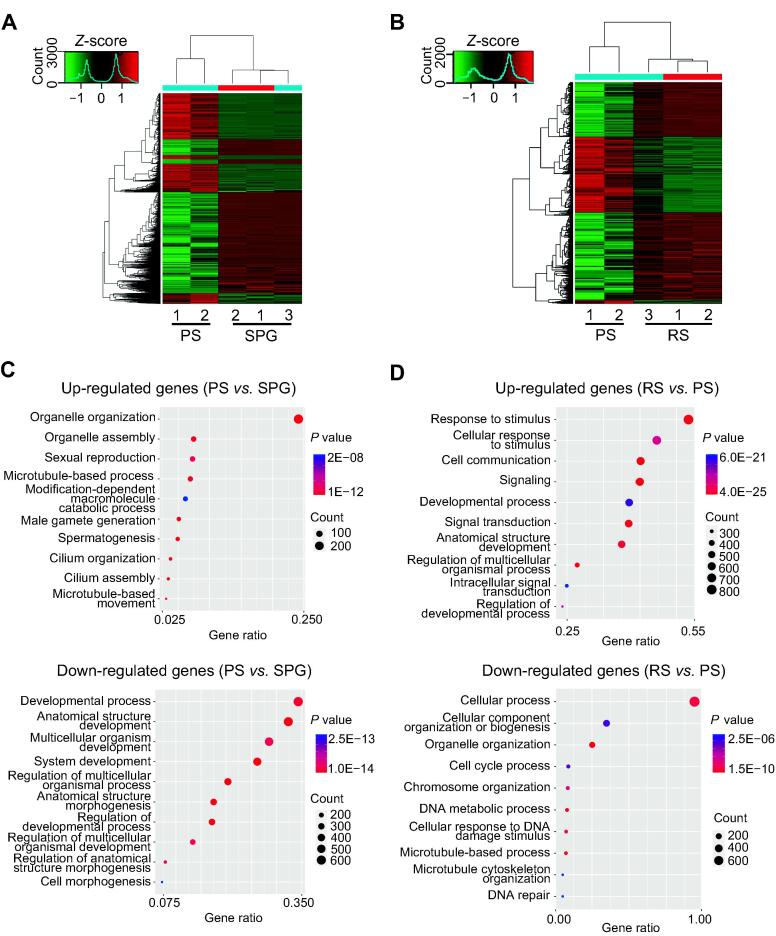
**Gene expression pattern during porcine spermatogenesis** **A.** The heatamap of DEGs between PS and SPG. **B.** The heatamap of DEGs between RS and PS. **C.** The enriched biological processes of DEGs between PS and SPG by GO analysis. **D.** The enriched biological processes of DEGs between RS and PS by GO analysis. Each dot plot shows gene ratio values of the top 10 significant enrichment terms. DEG, differentially expressed gene.

To further probe the regulatory roles of m^6^A, we performed RNA-seq analysis on these germ cells. We analyzed differentially expressed genes (DEGs; fold change ≥ 2, *P* < 0.05, FPKM ≥ 0.1) between continually developing germ cells. Results showed that 4393 and 5949 genes were up- and down-regulated in PS, respectively, when compared to SPG (Figure 4A; Table S2); 5119 and 2872 genes were up- and down-regulated in RS, respectively, when compared to PS (Figure 4B; Table S2). GO biological process annotation analysis revealed that in PS, the up-regulated genes (*vs.* SPG) mainly participated in cilium organization and spermatogenesis, while the down-regulated genes were involved in anatomical structure morphogenesis and regulation of developmental processes (Figure 4C). In RS (*vs.* PS), the up-regulated genes mainly regulated cell communication and developmental processes, whereas the down-regulated genes regulated chromosome organization and DNA metabolic process (Figure 4D). Given these findings on m^6^A-mediated processes (Figure 3C and D), we propose that the m^6^A modification might influence gene expression, thereby regulating spermatogenesis.

### m^6^A modification is involved in gene expression regulation

**Figure 5:**
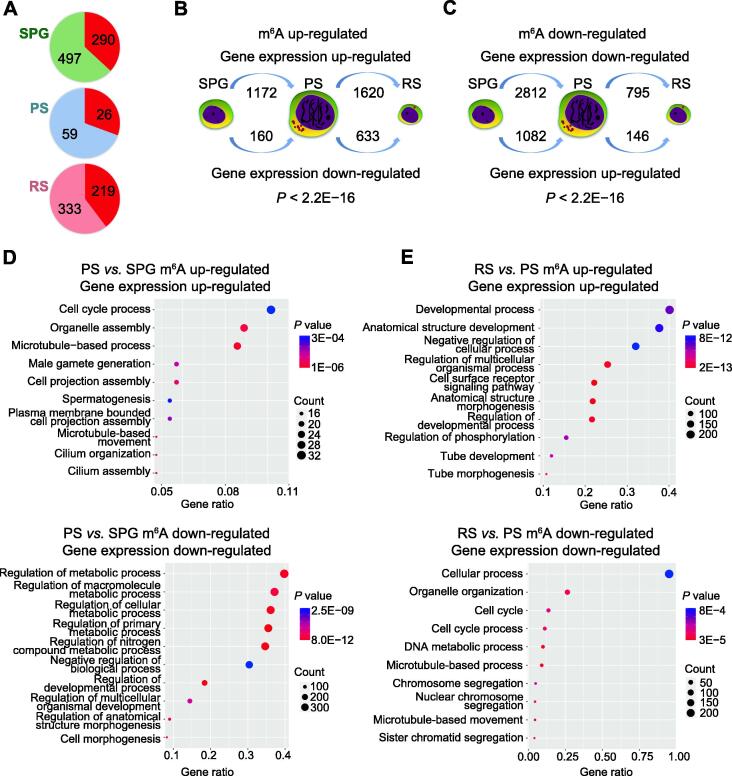
**m^6^A**-**regulatedgene expression during porcine spermatogenesis** **A.** The m^6^A modification distribution within stage-specific gene contexts. Number in the red part represents the number of genes specifically methylated in SPG, PS, or RS. **B.** and **C.** The number of up- or down-regulated genes during porcine spermatogenesis stratified by up-methylated (B) or down-methylated (C) genes. The *P* value of such difference was calculated with the Chi-square test. **D.** The enriched biological processes of m^6^A positively regulated genes between PS and SPG by GO analysis. **E.** The enriched biological processes of m^6^A positively regulated genes between RS and PS by GO analysis. Each dot plot shows gene ratio values of the top 10 significant enrichment terms.

We found that 36.8%, 30.6%, and 39.7% of the stage-specific transcripts, *i.e.*, for SPG, PS, and RS, respectively, were m^6^A modified (Figure 5A). To explore whether the m^6^A modification influences gene expression, we conducted a paired analysis of differentially methylated genes (DMGs) and DEGs between each two adjacent stages.

Among m^6^A up-methylated genes, 1172 and 1620 genes showed up-regulated expression during the transition from SPG to PS and from PS to RS, respectively (Figure 5B), while 160 and 633 genes showed down-regulated expression during the transition from SPG to PS and from PS to RS, respectively (Figure 5B). Among the m^6^A down-methylated genes, the expression levels of 2812 and 795 genes were decreased during the transition from SPG to PS and from PS to RS, respectively (Figure 5C), whereas those of 1082 and 146 genes were increased during the transition from SPG to PS and from PS to RS, respectively (Figure 5C). Hence, m^6^A exhibits positive correlation with gene expression.

To further uncover the biological significance of the dynamically modified m^6^A genes, we performed the GO biological process analysis with positive correlations with m^6^A modification. Compared to SPG, the up-regulated genes in PS mainly participated in the regulation of spermatogenesis and microtubule-based process (Figure 5D), and the down-regulated genes in PS were involved in the regulation of metabolic processes and developmental process (Figure 5E). Compared to PS, the up-regulated genes in RS regulated the developmental process and tube morphogenesis, and the down-regulated genes in RS participated in the sister chromatid segregation, DNA metabolic process, and microtubule-based process. Together, these findings reveal that m^6^A regulates gene expression and spermatogenesis.

### m^6^A-regulated gene expression is associated with the fate of SSCs

**Figure 6:**
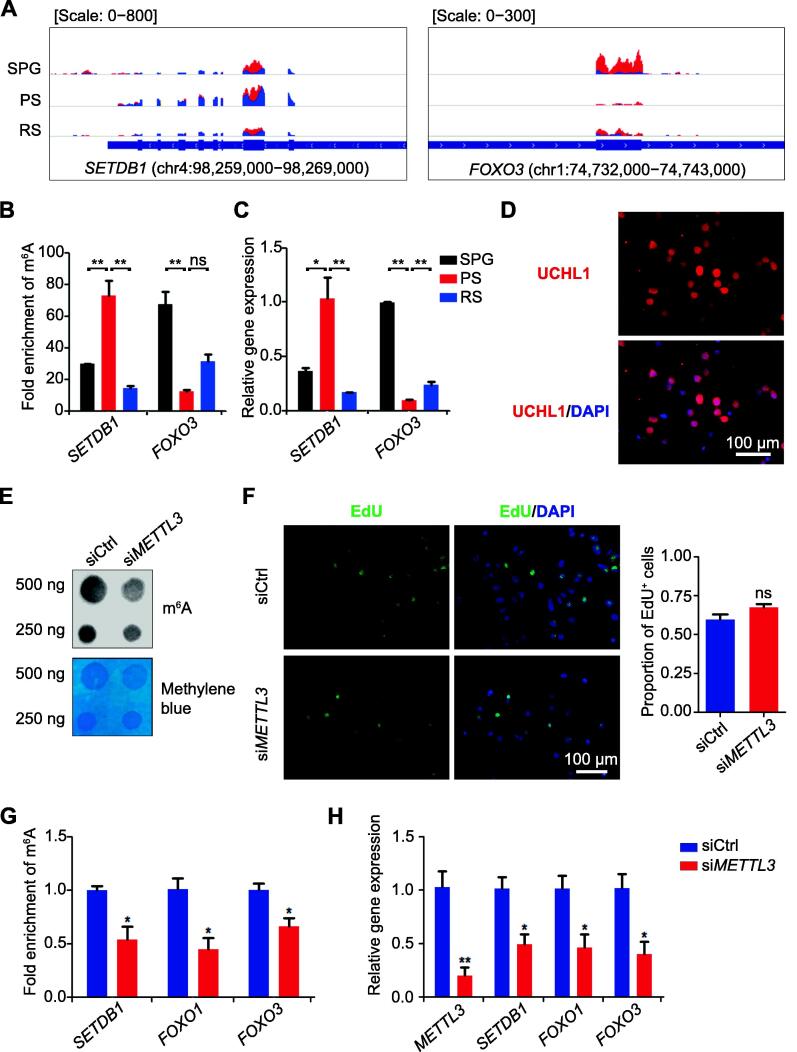
**Knockdown of *METTL3* in porcineSSCs induced the abnormal gene expression** **A.** Distribution of m^6^A on *SETDB1* and *FOXO3* during porcine spermatogenesis. Blue and red bars indicate the input and IP read coverage, respectively. **B.** Bar chart showing the m^6^A levels of *SETDB1* and *FOXO3* in SPG, PS, and RS validated by m^6^A-RIP-qPCR. **C.** Bar chart showing the mRNA levels of *SETDB1* and *FOXO3* in porcine SPG, PS, and RS validated by qRT-PCR. **D.** Immunocytochemistry showing the expression of UCHL1 in porcine SSCs. **E.** m^6^A dot blot analysis of the *METTL3* knockdown (si*METTL3*). siCtrl was used as a negative control. Methylene blue staining was used to evaluate RNA amount. **F.** Representative images of EdU incorporation in the cells transfected with si*METTL3* or siCtrl. The quantification analysis of EdU incorporation in the cells transfected with si*METTL3* or siCtrl was shown on the right. **G.** Bar chart showing the m^6^A levels of the targeted genes in the cells transfected with si*METTL3* or siCtrl validated by m^6^A-RIP-qPCR. **H.** Bar chart showing the relative expression level of *METTL3*, *SETDB1*, *FOXO1*, and *FOXO3* in the cells transfected with si*METTL3* or siCtrl detected by qRT-PCR. Data are presented as mean ± SEM. The *P* value was calculated with one-way ANOVA analysis followed by Bonferroni multiple-comparison test and unpaired *t*-test, *, *P* < 0.05; **, *P* < 0.01; ns, no significance. SSC, spermatogonial stem cell; IP, immunoprecipitation; EdU, 5-ethynyl-2′-deoxyuridine; SEM, standard error of mean. Scale bar, 100 µm.

Previous studies have shown that the methyltransferase SET domain bifurcated histone lysine methyltransferase 1 (SETDB1) catalyzes tri-methylation of histone H3 lysine 9 (H3K9me3) and plays important roles for SSC survival [25], [26], [27]. Meanwhile, deficiencies in FOXO1, FOXO3, and FOXO4 impair the self-renewal and differentiation of SSCs [28]. To determine whether m^6^A regulates the expression of these genes, we analyzed m^6^A modification patterns on *SETDB1* and *FOXO3* transcripts by m^6^A-RIP-qPCR. We found that both *SETDB1* and *FOXO3* transcripts contained at least one m^6^A peak (Figure 6A). *SETDB1* was up-methylated from SPG to PS and down-methylated from PS to RS, whereas *FOXO3* was down-methylated from SPG to PS and then up-methylated from PS to RS (Figure 6B). Quantitative real-time PCR (qRT-PCR) analysis revealed that the mRNA levels of *SETDB1* and *FOXO3* were strongly positively correlated with m^6^A modifications (Figure 6C).

To further validate the regulatory roles of m^6^A, we knocked down *METTL3* in porcine SSCs by a small interfering RNA (siRNA) [29], [30] (Figure 6D). Knockdown of *METTL3* (si*METTL3*) led to a decrease in m^6^A level, compared to the scramble control (siCtrl) (Figure 6E). Nevertheless, EdU incorporation showed that cell proliferation was not significantly different between cells transfected with siCtrl and si*METTL3* (Figure 6F). m^6^A-RIP-qPCR revealed that knockdown of *METTL3* significantly reduced the relative level of m^6^A in *SETDB1*, *FOXO1*, and *FOXO3* (Figure 6G). In addition, qRT-PCR analysis showed that the expression of these three targeted genes was significantly down-regulated (Figure 6H), consistent with the dynamics in SPG and PS showed by RNA-seq data (Figure 6A and C). Thus, these data suggest that m^6^A regulates the dynamic gene expression during porcine spermatogenesis.

## Discussion

Growing evidence has demonstrated the critical roles of m^6^A in murine spermatogenesis [11], [21]. The highly dynamic spermatogenesis process requires precise regulation of gene expression. Here, we reported that m^6^A modification was dynamically present in the transcripts of porcine male germ cells, which influenced gene expression.

In this study, m^6^A was distributed predominantly on the consensus motif of GGACU, which is consistent with murine male germ cells [23]. The m^6^A was present throughout mRNA transcripts in porcine germ cells, especially increased the read density in the CDS. The m^6^A reached its highest value around the stopC and then decreased in the 3′ UTR. This distribution pattern agrees with the previous findings in mouse [11]. Hence, m^6^A modification exhibits evolutionally conserved features in male germ cells in mouse and pig.

Note that the abundance of m^6^A in transcripts varied by the developmental stage during spermatogenesis. We found that m^6^A read density in the CDS was greater in PS and RS, whereas in the 3′ UTR it was greater in SPG. In a previous study, Lin et al. reported that m^6^A read density in the CDS in PS/diplotene spermatocytes and RS was higher than that in the undifferentiated SPG, type A1 SPG, and preleptotene spermatocytes [11]. The highest density of m^6^A reads was in the 3′ UTR close to the stopC of PS/diplotene spermatocytes, and the lowest of that was in type A1 SPG [11]. In addition, Tang et al. reported that m^6^A was enriched in long 3′ UTR transcripts of murine RS and elongating spermatids [22]. In general, m^6^A in the CDS is correlated with translational products, and m^6^A in the 3′ UTR is preferentially bound by factors regulating alternative splicing and polyadenylation, subcytoplasmic compartmentalization, and stability [31], [32]. Therefore, dynamically changed m^6^A around these landmarks could mediate specific transcript outputs in a stage-specific manner during mammalian spermatogenesis.

During spermatogenesis, SPG undergo mitosis to give rise to spermatocytes. Depletion of METTL3 or METTL14 in germ cells induced a 70% reduction of m^6^A in undifferentiated SPG and a 55%–65% reduction of m^6^A in PS [11]. METTL3 deficiency induced the abnormal initiation of spermatogonial differentiation and disrupted the ability of spermatocytes to reach the pachytene stage of meiotic prophase [21]. In this study, the down-methylated genes from SPG to PS mainly participated in metabolic processes, and most m^6^A down-methylated genes that were also mainly involved in metabolic and developmental processes were down-regulated. It is interesting that SPG that are localized in the basal compartment of seminiferous tubules exhibit high glycolytic activity [33]. In contrast, PS and RS, which are distributed in the luminal compartment, satisfy their ATP supply mainly through the aerobic (OXPHOS) pathway. Therefore, m^6^A might play critical roles in mediating mitochondrial function from SPG to PS.

A report showed that the loss of ALKBH5 markedly increased m^6^A levels in testes [4]. A delay in spermatocyte development occurred in the *ALKBH5*-knockout testes, which was due to the dysregulation of genes involved in meiotic progression [4], [22]. The up-methylated genes in PS preferentially participated in spermatogenesis and cell cycle process. These genes were also up-regulated by m^6^A modification, indicating an important role of m^6^A at early developmental stages. To generate the haploid spermatids, spermatocytes must undergo two meiotic divisions. The first meiotic division promotes the pairing and exchange of genetic materials, and the second meiotic division is more comparable to mitotic divisions as it contains the segregation of sister chromatids [34]. In addition, m^6^A in PS was also enriched in genes that functioned in spermatid development and up-regulated the expression of genes involving in microtubule-based process, cilium organization, and cilium assembly. It is reasonable to speculate that m^6^A-methylated mRNAs may repress translation before spermiogenesis.

During spermiogenesis, RS go through multistep cytological changes, such as the formation of an acrosome and a flagellum, chromatin remodeling, and the removal of the residual body [15], [35]. *METTL3*-knockout or *METTL14*-knockout result in a 45% reduction of m^6^A abundance in RS [11]. The seminiferous tubules contain very few spermatozoa, and sperms exhibit defects in motility, flagella, and head [11]. In the present study, m^6^A up-methylated genes in RS showed up-regulated expression, and these genes mainly participated in regulating developmental process, including the development of multicellular organisms, anatomical structures, and tube morphogenesis, suggesting the conserved roles of m^6^A in mediating porcine spermiogenesis. At the beginning of spermiogenesis, nuclear condensation begins and histones are rapidly replaced by protamines [36], [37]. The mRNAs are massively eliminated during spermiogenesis [38]. m^6^A in PS shows heavy enrichment in genes regulating chromosome organization and nucleic acid metabolic, which are essential for meiosis and down-regulated in RS. Because of the greater m^6^A retention in the longer pre-mRNA of the *ALKBH*-knockout testis, the splicing of these transcripts is enhanced and further causes the production of shorter transcripts. m^6^A modification of the short transcripts further serves as a signal to quickly degrade elongating spermatids [22]. Piwi-interacting RNAs (piRNAs) are responsible for degrading large populations of mRNAs in the late stage of spermiogenesis [38]. It is intriguing that piRNA target sites are preferentially located in the 3′ UTR of the target mRNAs [39]. Given the high-level m^6^A modification on the mRNA 3′ UTR, it is possible that m^6^A mediates the piRNA-dependent mRNA degradation pathway during spermiogenesis.

Expression of *FOXOs* in germ cells is intimately associated with cell fate, which are responsible for cell cycle arrest and programmed cell death [40]. Our previous studies revealed that knockdown of *SETDB1* to an extremely low level could activate *FOXO1* that is the most important *FOXOs* in spermatogenesis [25], [26], [27]. Here, we provide evidence that m^6^A regulates the expression of *SETDB1*, *FOXO1*, and *FOXO3* during porcine spermatogenesis. Unlike the hypo-proliferation in *SETDB1*-knockdown SSCs or gonocytes [26], [27], *METTLE3*-knockdown mildly promotes proliferation, suggesting that other methyltransferases, such as SUV39H1 and SUV39H2, may partially compensate for SETDB1 deficiency [41]. Moreover, deleting *METTL3* from murine SSCs induces the hyper-proliferation and impaired differentiation [11], [21]. Recent work has showed that m^6^A on retroviral element RNAs recruit SETDB1 to regulate heterochromatin in mouse embryonic stem cells [42], [43]. It would be worthwhile to study whether and how m^6^A regulates *SETDB1* expression and H3K9 methylation, which further govern the fate of SSCs.

In conclusion, we present here the first m^6^A transcriptome-wide map of porcine spermatogenesis. Our findings provide a roadmap for uncovering m^6^A functions that might improve porcine fertility and treat infertility in humans.

## Materials and methods

### Animals

Testis samples of 5-month-old pigs were acquired from the Besun farm, Yangling, China. After the castration, testes were transported to the pre-cold PBS (4 °C) contained 2% of penicillin and streptomycin (Catalog No. SV30010, HyClone, Logan, UT) and brought to the laboratory within 2 h. Testes were cut into small pieces and subjected to two-step enzyme digestion as previously described [44]. After digestion with collagenase Type IV (0.2% w/v; Catalog No. 17104019, Gibco, Grand Island, NY) at 37 °C for 30 min, the obtained seminiferous tubules were further digested by 0.25% trypsin–EDTA (Catalog No. SV30031.01, HyClone) at 37 °C for 15 min. Then, the germ cells were collected by the differential plating.

The isolation of SPG, PS, and RS was conducted as previously described [45]. In brief, 1 × 10^7^–1 × 10^8^ dispersed germ cells were suspended in 50-ml DMEM plus 0.5% BSA and load onto a 600-ml gradient of 2%–4% BSA for 3 h sediment at 4 °C. Approximately 100 6-ml fractions were collected in tubes and analyzed by the morphology and immunostaining. Fractions with high purity of SPG, PS, and RS were resuspended with TRIzol (Catalog No. 15596026, Invitrogen, Vilnius, Lithuania) and stored at −80 °C until usage.

### LC-MS/MS quantification of m^6^A levels

LC-MS/MS quantification of m^6^A was performed by Cloudseq Biotech Inc. (Shanghai, China) following the vendors recommended protocol. Total RNA was isolated using TRIzol reagent (Catalog No. 15596026, Invitrogen) following to the manufacturer’s instruction. In brief, 1 µg of total RNA was digested by 4-µl nuclease P1 (Catalog No. N8630, Sigma, St. Louis, MO) in 40-µl buffer solution (10 mM Tris-HCl pH 7.0, 100 mM NaCl, 2.5 mM ZnCl_2_) at 37 °C for 12 h, followed by incubating with 1-µl alkaline phosphatase (Catalog No. P5931, Sigma) at 37 °C for 2 h. RNA solution was diluted to 100 µl and injected into LC-MS/MS. The nucleosides were separated by reverse phase high-performance liquid chromatography on an Agilent C18 column (Catalog No. 5188–5328, Agilent Technologies, San Diego, CA), coupled with MS detection using AB SCIEX QTRAP 5500 (Catalog No. AB Sciex QTrap 5500, AB Sciex LLC, Framingham, MA). Pure nucleosides were used to generate standard curves, from which the concentrations of adenosine (A) and m^6^A in the sample were calculated. The level of m^6^A was then calculated as a percentage of total unmodified A.

### Cell culture and RNA-interference-mediated ***METTL3*** knockdown

The porcine SSC line was cultured in the complete medium made up of DMEM (high glucose, pyruvate; Catalog No. 11995065, Gibco), 5% (v/v) fetal bovine serum (FBS; Catalog No. 12664025, Gibco, Mesenchymal Stem Cell FBS Qualified), 5% (v/v) knockout serum replacement (KSR; Catalog No. 10828028, Gibco), 2 mM Glutamax (Catalog No. 35050061, Gibco), 1× MEM Non-Essential Amino Acids Solution (Catalog No. 11140076, Gibco), 1× MEM Vitamin Solution (Catalog No. 11120052, Gibco), 5 × 10^−^^5^ M 2-mercaptoethanol (Catalog No. M6250, Sigma), 1× penicillin–streptomycin (Catalog No. SV30010, HyClone), 20 ng/ml recombinant human GDNF (Catalog No. 45010, PeproTech, Rocky Hill, NJ), 40 ng/ml recombinant human GFRA1 (Catalog No. 788104, BioLegend, San Diego, CA), and 10 ng/ml recombinant human bFGF (Catalog No. 10018B, PeproTech). The cells were maintained at 37 °C in the presence of 5% CO_2_. For *METTL3* knockdown, porcine SSCs were transfected with 50 pM of siRNA duplexes against porcine *METTL3* (GenePharma, Shanghai, China; the RNA oligos are listed in Table S3), using Advanced DNA RNA Transfection Reagent (Catalog No. AD600025, ZETA LIFE, Menlo Park, CA) in antibiotic-free medium. Cells were collected for analysis 72 h after transfection. The cells were lysed by TRIzol (Catalog No. 15596026, Invitrogen) and stored at −80 °C until usage.

### Immunocytochemistry

The isolated SPG, PS, and RS were fixed with 4% paraformaldehyde for 25 min at 4 °C and washed with PBS for three times. Then, the cells were permeabilized for 10 min using 0.1% Triton-X 100 followed by washing with PBS for three times. The cells were further blocked with 10% donkey serum for 2 h at room temperature, and incubated with primary antibodies, including UCHL1 (Catalog No. ab8189, Abcam, Cambridge, Britain), SYCP3 (Catalog No. ab15093, Abcam), and CD63 (Catalog No. 25682-1-AP, Proteintech, Wuhan, China) at a dilution with 1:200 overnight at 4 °C. Next day, the cells were washed with PBS for 4 times and incubated with secondary antibody (Yeasen, Shanghai, China) at a dilution with 1:400 for 1 h at room temperature. For cell proliferation detection, porcine SSCs were detected for the EdU incorporation by Cell-Light EdU Apollo488 *in vitro* Kit (Catalog No. C10310-3, RiboBio, Guangzhou, China) according to the manufacturer’s protocol. The nucleus was labeled with DAPI (Catalog No. BD5010, Bioworld Technology, St. Louis Park, MN). A fluorescence microscope (Leica, Germany) was used for fluorescence observation and photographing.

### m^6^A-RIP-seq and data analysis

m^6^A-RIP-seq was performed by Cloudseq Biotech Inc. (Shanghai, China) as previously described [46]. In brief, total RNA was extracted by using TRIzol (Catalog No. 15596026, Invitrogen). Denaturing agarose electrophoresis was used for confirming RNA integrity. Then, mRNA was isolated from total RNA by Seq-Star poly(A) mRNA Isolation Kit (Catalog No. AS-MB-006-01, Arraystar, Rockville, MD). The m^6^A RNA immunoprecipitation (IP) was conducted by GenSe m^6^A RNA IP Kit (Catalog No. GS-ET-001, GenSeq, Shanghai, China) according to the manufacturer’s instructions. Libraries were constructed from the samples with and without m^6^A IP by NEBNext Ultra II Directional RNA Library Prep Kit (Catalog No. E7760, New England Biolabs, Ipswich, MA). Library was evaluated by the BioAnalyzer 2100 system (Catalog No. G2939BA, Agilent Technologies) and sequenced on an Illumina Hiseq 4000 (Catalog No. SY-401-4001, Illumina, San Diego, CA) with 150 bp paired-end reads.

After quality controlled by Q30, 3′ adaptor and low-quality reads were removed by cutadapt software (v1.9.3). Clean reads of all libraries (*n* = 3 for each group) were aligned to the reference genome (susScr11) by Hisat2 software (v2.0.4; −*p* 10 −q −−rna−strandness RF). Methylated sites were identified by MACS (v1.4) software (*P* < 0.00001) and visualized by Integrative Genomics Viewer (IGV; v2.5.0). Motifs enriched in m^6^A peaks were identified using DREME [47]. Fifty nucleotides on each side of the top 1000 peaks in each sample were used for motif enrichment. GO analysis was performed by R package topGo (v3.2). The dot plot shows the gene ratio values of the top 10 significant enrichment terms.

### Dot-blot

RNA was isolated and loaded on the positively charged nylon transfer membrane. After crosslinking by UV, the membrane was blocked by the 5% non-fat milk for 2 h, and followed by incubation with rabbit anti-m^6^A antibody (1:1000; Catalog No. 202003, Synaptic Systems, Göttingen, Germany) at 4 °C overnight. Then, the membrane was incubated with HRP-conjugated goat anti-rabbit IgG at room temperature for 2 h followed by ECL imaging system (Catalog No. WBKLS0100, Millipore, Burlington, MA). Finally, the membrane was stained with 0.02% methylene blue to evaluate RNA amount.

### RNA-seq and data analysis

The rRNAs were removed from total RNA by NEBNext rRNA Depletion Kit (Catalog No. E6310, New England Biolabs) following the manufacturer’s instruction. NEBNext Ultra II Directional RNA Library Prep Kit (Catalog No. E7760, New England Biolabs) was used to construct RNA libraries. Library quality was confirmed by BioAnalyzer 2100 system (Catalog No. G2939BA, Agilent Technologies). Library sequencing was performed on an Illumina Hiseq 4000 (Catalog No. SY-401-4001, Illumina) with 150 bp paired end reads.

After raw data process (*n* = 3 for each group), high-quality clean reads were aligned to the reference genome (susScr11) with Hisat2 software (v2.0.4; −*p* 10 −q −−rna−strandness RF). Then, HTSeq software (v0.9.1) was used to get the raw count, and edgeR was used to perform normalization based on the Ensembl gtf gene annotation file (v11.1.103). The differentially expressed mRNA was identified and used for further analysis. The R package heatmap.2 was used for heat-map drawing.

### m^6^A-RIP-qPCR

Total RNA was extracted from porcine SPG, PS, and RS using TRIzol (Catalog No. 15596026, Invitrogen). mRNA was isolated using the PolyATtract mRNA Isolation Systems (Catalog No. Z5310, Promega, Madison, WI) following the manufacturer’s instructions. IP mixture was composed by 6 µg of rabbit anti-m^6^A antibody (Catalog No. 202003, Synaptic Systems), mRNA, IP buffer (50 mM Tris-HCl pH 7.4, 750 mM NaCl, and 0.5% NP-40), RNase inhibitor (Catalog No. AM2682, Invitrogen), and RNase-free water up to 500 µl in total volume. After being mixed by rotating for 2 h at 4 °C, the IP mixture was incubated with the Protein A beads (Catalog No. 10002D, Invitrogen) which have been washed for three times and blocked by 0.5 mg/ml BSA, followed by rotating overnight at 4 °C. Precipitated mRNA was eluted using elution buffer (1× IP buffer, 6.7 mM m^6^A). For the detection of the fold enrichment of m^6^A level, precipitated mRNA and input RNA were subjected to cDNA synthesis and qRT-PCR, respectively. The primers are listed in Table S3.

### qRT-PCR

qRT-PCR analysis was performed with FastStart Essential DNA Green Master (Catalog No. 06402712001, Roche, Mannheim, Germany) using an IQ-5 (Bio-Rad, Hercules, CA). The relative expression was normalized to *GAPDH* and *HPRT1*, and then calculated using the comparative Ct method (2^−ΔΔC^^t^). The primers are listed in Table S3.

## Ethical statement

All experimental procedures involving animals were approved by the Northwest A&F University’s Institutional Animal Care and Use Committee, China (Approval No. DK-20180375).

## Data availability

The datasets generated in the current study have been deposited in the Genome Sequence Archive [48] at the National Genomics Data Center, Beijing Institute of Gemonics, Chinese Academy of Sciences / China National Center for Bioinformation (GSA: CRA003615), and are publicly accessible at https://ngdc.cncb.ac.cn/gsa.

## Competing interests

The authors have declared no competing interests.

## CRediT authorship contribution statement

**Zidong Liu:** Conceptualization, Validation, Formal analysis, Investigation, Writing – original draft, Visualization. **Xiaoxu Chen:** Conceptualization, Formal analysis, Investigation, Writing – original draft, Visualization. **Pengfei Zhang:** Validation, Investigation. **Fuyuan Li:** Validation, Investigation. **Lingkai Zhang:** Formal analysis. **Xueliang Li:** Writing – review & editing. **Tao Huang:** Investigation, Writing – original draft. **Yi Zheng:** Writing – review & editing. **Taiyong Yu:** Resources. **Tao Zhang:** Conceptualization, Funding acquisition. **Wenxian Zeng:** Conceptualization, Writing – review & editing, Supervision, Project administration, Funding acquisition. **Hongzhao Lu:** Writing – review & editing. **Yinghua Lv:** Writing – review & editing, Funding acquisition. All authors have read and approved the final manuscript
