# Supplementary material for: Transcriptome-wide Dynamics of m6A mRNA Methylation During Porcine Spermatogenesis
Source: Genomics Proteomics Bioinformatics. 2021 Sep 17;21(4):729–41. doi: 10.1016/j.gpb.2021.08.006 (PMC10787014; doi:10.1016/j.gpb.2021.08.006)
Supplement: Supplementary Table S3 [file mmc3.docx]

| **Oligo name** | **Sequences (5'–3')** |
| --- | --- |
| *SETDB1*-q-F | ATCCCATTTGCCGACCACTAA |
| *SETDB1*-q-R | ACCATTGGGCGGTTTGGATAG |
| *FOXO1*-q-F | CCATGCTACTCATTTGCGCC |
| *FOXO1*-q-R | GAGTCCCGCTGCACAGTTAT |
| *FOXO3*-q-F | CTGCCGGCTGGAAGAACTCTA |
| *FOXO3*-q-R | GCTCTTTCCCCCATCAGGGT |
| *ACTB*-q-F | TGGAACGGTGAAGGTGACAG |
| *ACTB*-q-R | CTTTTGGGAAGGCAGGGACT |
| *GAPDH*-q-F | GTTTCCTCGTCCCGTAGACA |
| *GAPDH*-q-R | TCGTTGATGGCAACAATCTC |
| *HPRT1*-q-F | GAAGAGCTACTGTAATGACCAGTCAACGG |
| *HPRT1*-q-R | TCATTGTAGTCAAGGGCATAGCCTACC |
| *SETDB1*-rip-F | CTATTGCAGCTCAACCACGA |
| *SETDB1*-rip-R | TTCCTGTCTCCCCATCAATC |
| *FOXO1*-rip-F | GGATCGACTGGCGTCATAAT |
| *FOXO1*-rip-R | TGCAAACACTTCAGGACAGC |
| *FOXO3*-rip-F | ACAGTGTTTGGACCCTCGTC |
| *FOXO3*-rip-R | CAGGTCCTGGAGTGTCTGGT |
| *siMETTL3*-sense | ACUUCUUCUCUAAUUCAGGGU |
| *siMETTL3*-anti | ACCCUGAAUUAGAGAAGAAGU |
| siCtrl-sense | UUCUCCGAACGUGUCACGUTT |
| siCtrl-anti | ACGUGACACGUUCGGAGAATT |

**Table S3 Oligo information**
